# Comment on Devoogdt et al. The Effectiveness of Fluoroscopy-Guided Manual Lymph Drainage as Part of Decongestive Lymphatic Therapy on the Superficial Lymphatic Architecture in Patients with Breast Cancer-Related Lymphoedema: A Randomised Controlled Trial. *Cancers* 2023, *15*, 1545

**DOI:** 10.3390/cancers16132434

**Published:** 2024-07-02

**Authors:** Pierre Bourgeois

**Affiliations:** Services of Nuclear Medicine, Institut Jules Bordet and Hôpitaux Iris Sud—Iris Ziekenhuizen Zuid (HIS-IZZ) Hospitals, Université Libre de Bruxelles, 1070 Brussels, Belgium; pierre.bourgeois@hubruxelles.be

This study is a comment on the non-effectiveness of fluoroscopy-guided manual lymph drainage using ICG as part of decongestive lymphatic therapy on the superficial lymphatic architecture in patients with breast cancer-related lymphoedema in a randomized controlled trial, and the demonstration of one toxicity of indocyanine green on the lymphatic system.

We read with interest the article by Devoogdt et al. entitled “The Effectiveness of Fluoroscopy-Guided Manual Lymph Drainage as Part of Decongestive Lymphatic Therapy on the Superficial Lymphatic Architecture in Patients with Breast Cancer-Related Lymphoedema: A Randomised Controlled Trial” [1].

The authors used ICG to highlight superficial lymphatic structures of edematous upper limbs secondary to breast cancer surgery and to study changes in the areas of dermal reflux, and the number of vessels and lymph nodes in three groups of patients subjected to different physiotherapeutic protocols (a “placebo” group, a group with traditional MLD and a group with fluoro-guided MLD). They compared the basal situation to those observed after 3 weeks of intensive treatment and 6 months of maintenance treatment. Their initial hypothesis was that patients receiving fluoro-guided drainage would demonstrate an increase in the number of lymphatic vessels (LV) draining the areas of dermal reflux, a decrease in these areas of reflux and an increase in the number of lymph nodes (LN) receiving lymph from these areas of lymphatic stasis. Their results show no difference between their three groups, and they conclude that MLD has no benefit over the other components of DLT. However, they also observed a significantly decreased number of LV in their group with traditional MLD after their intensive phase (and borderline after their maintenance phase) and, in the whole group, a significant decrease in dermal backflows and in the number of LN visualized (both after their intensive phase and after their maintenance phase). Their results raise an otherwise important problem that would question their main conclusion (an absence of effect of MLD, among other factors, on the reduction in edema volumes), and more generally their approach, namely, due to a potential toxic effect of ICG on the lymphatic system.

At first glance, the limited data in the literature on the subject appear to be somewhat contradictory.

Aldrich et al. [2] were unable to detect changes in LV function from the inguinal LN to the axillary LN in mice within the minute after (free) ICG injections in various concentrations, but at a given time and no later than two hours after injection.

Gashev et al. [3], on the other hand, showed that, on ex vivo isolated rat mesenteric LV, ICG (diluted in a physiological NaCl solution containing albumin) binds to the endothelial cell layer and inhibits the contraction of these vessels in a dose-dependent manner (and continues to modify function even beyond complete vessel washing after a delay of several minutes).

Weiler and Dixon’s study [4] appears to be interesting in this context. These authors analyzed in vivo the consequences on the function of rat tail LV (at 1, 2 and 4 weeks) of ICG injection (pre-mixed with BSA) and its consequences on the LN accumulating this tracer. ICG remained visible for up to 2 weeks after injection and was accompanied (compared to controls) by significant decreases in LV function at week 1 and enlargement of the LN draining this ICG (with an increase in size of more than 350% at week 1 and nearly 200% at week 2). The influence of ICG injection on the lymphatic system therefore seems to be delayed rather than immediate and seems to require a long pre-exposure to the molecule to show an effect.

The toxicity of ICG has been widely studied both in vitro and in vivo by ophthalmologists who have long had a common use of it, and a reading of their literature on the subject leads to the following definitive conclusion: ICG shows a dose-dependent and time exposure-dependent toxicity [5]. This is supported by clinical data in humans, because better functional outcomes were obtained when low dye concentrations and short incubation times are reported.

The decrease in dermal reflux, lymphatic vessel count and lymph node numbers observed by Devoogdt et al. could therefore reflect a decrease in the function of the lymphatic system, of the LV to extract ICG from injected tissues and to transport it to and into the LN.

As things stand, the conclusions of their study (absence of effects and interest of manual lymphatic drainage in the context of DLT) seem to us to be at the very least questionable (even when fluoro-guided, because the procedure would then concentrate the possible toxicity of ICG on lymphatic structures).
